# Hydroxylated Metabolites of Polybrominated Diphenyl Ethers in Human Blood Samples from the United States

**DOI:** 10.1289/ehp.11660

**Published:** 2008-08-01

**Authors:** Xinghua Qiu, Robert M. Bigsby, Ronald A. Hites

**Affiliations:** 1 School of Public and Environmental Affairs, Indiana University, Bloomington, Indiana, USA;; 2 Department of Obstetrics and Gynecology, School of Medicine, Indiana University, Indianapolis, Indiana, USA

**Keywords:** bromophenols, flame retardants, HO-PBDEs, human blood, metabolites, PBDEs, polybrominated diphenyl ethers

## Abstract

**Background:**

A previous study from our laboratory showed that polybrominated diphenyl ethers (PBDEs) were metabolized to hydroxylated PBDEs (HO-PBDEs) in mice and that *para*-HO-PBDEs were the most abundant and, potentially, the most toxic metabolites.

**Objective:**

The goal of this study was to determine the concentrations of HO-PBDEs in blood from pregnant women, who had not been intentionally or occupationally exposed to these flame retardants, and from their newborn babies.

**Methods:**

Twenty human blood samples were obtained from a hospital in Indianapolis, Indiana, and analyzed for both PBDEs and HO-PBDEs using electron-capture negative-ionization gas chromatographic mass spectrometry.

**Results:**

The metabolite pattern of HO-PBDEs in human blood was quite different from that found in mice; 5-HO-BDE-47 and 6-HO-BDE-47 were the most abundant metabolites of BDE-47, and 5′-HO-BDE-99 and 6′-HO-BDE-99 were the most abundant metabolites of BDE-99. The relative concentrations between precursor and corresponding metabolites indicated that BDE-99 was more likely to be metabolized than BDE-47 and BDE-100. In addition, three bromophenols were also detected as products of the cleavage of the diphenyl ether bond. The ratio of total hydroxylated metabolites relative to their PBDE precursors ranged from 0.10 to 2.8, indicating that hydroxylated metabolites of PBDEs were accumulated in human blood.

**Conclusions:**

The quite different PBDE metabolite pattern observed in humans versus mice indicates that different enzymes might be involved in the metabolic process. Although the levels of HO-PBDE metabolites found in human blood were low, these metabolites seemed to be accumulating.

Polybrominated diphenyl ethers (PBDEs) are widely used as flame retardants in polyurethane foams, textiles, and electric appliances. The annual global sales of PBDEs were around 67,100 metric tons in 1999 and 67,400 metric tons in 2001 (Bromine Science and Environmental Forum 2006). Because PBDEs are very stable and are not chemically bonded to the material in which they are used, they are widely found in the environment and have also been found in human tissue (Hites 2004; Law et al. 2006). In human tissue, the tetra-, penta-, and hexabrominated congeners, especially BDEs 47, 99, 100, 153, and 154, were more predominant than the more highly brominated congeners, such as BDE-209 (Hites 2004). As a result of their environmental ubiquity, two commercial PBDE products, containing tetra- through octabrominated congeners, have been banned by the European Union and by several U.S. states, and the major manufacturer of these two products in the United States stopped producing them in 2004 (Renner 2004).

The prevalence of PBDEs in human tissue is of concern because of their potential toxicity; these effects include carcinogenicity, neurotoxicity, reproductive toxicity, and thyroid toxicity (Darnerud et al. 2001). Although the toxicity of PBDEs is not fully understood, some of the toxic effects might be due to their hydroxylated metabolites, especially the hydroxylated PBDEs (HO-PBDEs). For instance, levels of serum thyroxine (T_4_, a thyroid hormone and the precursor of active thyronine, T_3_) were significantly decreased when rats were exposed to PBDEs (Darnerud et al. 2007; Stoker et al. 2004; Zhou et al. 2002). The effect of PBDEs on T_4_ levels may require metabolic activation because HO-PBDEs, but not the PBDE congeners themselves, behave as ligands for human transthyretin (TTR; a major thyroid hormone transport protein) *in vitro* (Meerts et al. 2000). In addition, Hamers et al. (2008) reported that the transthyretin-binding potencies of HO-PBDEs were orders of magnitude higher than that of BDE-47. Similarly, PBDE has mild estrogenic effects in mice (Mercado-Feliciano and Bigsby 2008a), and these effects are likely caused by HO-PBDEs that act as ligands for the estrogen receptor (Mercado-Feliciano and Bigsby 2008b). In addition, HO-PBDEs were shown to inhibit estradiol-sulfotransferase (Hamers et al. 2008) and placental aromatase (Cantón et al. 2008).

HO-PBDEs have been identified in blood samples from rats and mice after exposure to PBDE mixtures (Malmberg et al. 2005; Qiu et al. 2007) and observed in blood samples from wild animals such as fishes, birds, and mammals (Marsh et al. 2004; Valters et al. 2005; Verreault et al. 2005). However, with one exception, there have been no reports about PBDE metabolites in human blood. The exception was a report about HO-PBDEs in pooled human blood samples taken from children living or working at a municipal waste disposal site in Managua, Nicaragua (Athanasiadou et al. 2008). To study the metabolism of PBDEs in humans, we have identified and quantitated the hydroxylated metabolites of PBDEs, including HO-PBDEs and bromophenols, in 20 individual human blood samples from pregnant women and newborn babies from the United States. HO-PBDEs and bromophenols were both found to be important metabolites of PBDEs in plasma after mice were exposed to a commercial PBDE mixture (Qiu et al. 2007). Here we report these HO-PBDE concentrations and compare these levels to those measured in mouse blood after exposure to high levels of DE-71, a commercial penta-BDE mixture (Qiu et al. 2007).

## Materials and Methods

### Chemicals

4′-HO-BDE-17, 2′-HO-BDE-28, 4-H O - B D E - 4 2, 3 -H O -B D E -4 7, 5- H O - B D E - 4 7, 6 -H O -B D E -4 7, 4′-HO-BDE-49, 2′-HO-BDE-66, and 2′-HO-BDE-68 were gifts from Göran Marsh (Stockholm University, Stockholm, Sweden) and were synthesized as described elsewhere (Marsh et al. 2004). 4-MeO-BDE-90, 6′-MeO-BDE-99, and 2,4,6-tribromophe -nol were from AccuStandard (New Haven, CT). 5′-MeO-BDE-99, 5′-MeO-BDE-100, 4′-MeO-BDE-101, 4′-MeO-BDE-103, and 4-HO-^13^C_12_-PCB-187 were from Wellington Laboratories (Guelph, ON, Canada). 2,4-Dibromophenol and 2,4,5-tribromophenol were from Cambridge Isotope Laboratories (Cambridge, MA). All the PBDE congeners (BDE-28, 47, 71, 77, 99, 100, 153, 154, and 166) were purchased from AccuStandard. The full names of these compounds are listed in the Supplemental Material (available online at http://www.ehponline.org/members/2008/11660/suppl.pdf).

All the phenolic compounds were methylated with fresh diazomethane, which was prepared from Diazald (Sigma Chemical Co., St. Louis, MO) (Black 1983). All the organic solvents and water used for the extraction and cleanup procedures were residue-analysis grade.

### Sample collection

Human studies were performed in accordance with the guidelines and approval of the Institutional Review Board at Indiana University School of Medicine. Pregnant women were enrolled in the study during 2003–2004 at Wishard Memorial County Hospital (Indianapolis, IN). Random fetal blood samples (*n* = 16) were collected from the umbilical cord vein by syringe after delivery; these samples were not accompanied by any clinical data or other information about the pregnant women. Upon consent to enter the study, four women were administered a questionnaire to determine potential sources of contamination; none of the subjects had any identifiable source of occupational exposure to PBDE. Maternal blood was obtained upon admission to the maternity ward; in this study, only one of the four maternal samples was matched with a fetal sample. Blood samples were collected in heparinized tubes, maintained at 4°C, and centrifuged at 800 × *g* for 15 min to allow collection of the plasma fraction. All the plasma samples were kept at −20°C until extraction.

### Sample extraction and preparation

We slightly modified previous methods (Hovander et al. 2000; Qiu et al. 2007) for this study. Twenty samples (from 4.2 g to 13.8 g, with an average wet weight of 8.9 g) were analyzed. Before extraction, each sample was transferred to a clean centrifuge tube and spiked with a known amount of BDE-77 and 4-HO-^13^C_12_-PCB -187 as recovery surrogate standards. Hydrochloric acid (1 mL, 6 M) and 2-propanol (6 mL) were added; the sample was vortexed after each addition. After denaturizing the samples, they were extracted three times, each time with 6 mL of a hexane/ methyl *tert*-butyl ether mixture (1:1 by volume). The organic extracts were combined, and approximately 20% of the solution was removed for gravimetric determination of lipid mass. The rest of each sample was blown down to 2 mL with clean nitrogen, and 2 mL of potassium hydroxide (0.5 M in 50% ethanol) was added to ionize the phenolic analytes. After extraction with hexane three times to separate the PBDEs, the aqueous phase was acidified with hydrochloric acid (2.1 mL, 0.5 M), then the phenolic compounds were extracted three times with a hexane/methyl *tert*-butyl ether mixture (9:1 by volume).

The neutral fraction was treated with 5 mL of concentrated sulfuric acid twice to remove lipids, followed by alumina column chromatography (6 cm × 0.6 cm i.d., with 0.5 cm anhydrous sodium sulfate on the top). The column was eluted with 8 mL of hexane, followed by 8 mL of a hexane/dichloromethane mixture (3:2 by volume). The PBDE congeners were in the second fraction. BDE-71 was added as an internal standard, and the samples were blown down to approximately100 μL before gas chromatographic mass spectrometry (GC/MS) analysis.

We concentrated the phenolic fraction by nitrogen blow-down, and the residual water was removed with an anhydrous sodium sulfate column (5 cm × 0.6 cm i.d.). To methylate the phenolic analytes, samples were treated with diazomethane at room temperature overnight. After methylation, the samples were treated with concentrated sulfuric acid three times to remove lipid, followed by alumina column chromatography, which was the same as that used for the neutral fraction. Finally, BDE-166 was added as the internal standard, and samples were blown down to approximately 50 μL for GC/MS analysis. To prevent potential photo-degradation, during the whole process the centrifuge tubes were wrapped with aluminum foil, or amber vials were used.

### Instrumental analysis

We analyzed both neutral and methylated phenolic fractions by GC/MS (Agilent 6890/5973) with an electron-capture-negative ionization (ECNI) ion source. We used selected ion monitoring of *m/z* 79 and 81 for quantitation. The GC injection port was held at 285°C, with an injection volume of 2 μL. A non-polar Rxi-5ms column (15 m length; 250 μm i.d.; 0.25 μm film thickness; Restek Corp., Bellefonte, PA) was used to separate both the neutral and methylated phenolic analytes. The GC oven temperature program was as follows: held at 60°C for 1 min; 10°C/ min to 240°C; 25°C/min to 325°C; held for 8 min. The same instrument, but with a polar SP-2331 column (30 m length; 250 μm i.d.; 0.20 μm film thickness; Supelco Inc., Bellefonte, PA), was used for the confirmation of the methylated phenolic analytes. In this case, the GC temperature program was as follows: held at 80°C for 1 min; 10°C/min to 260°C; held for 16 min. Method detection limits were 0.5–2 pg/g plasma.

### Quality control

Several quality control criteria were used to ensure the correct identification and quantitation of the target compounds. First, the GC retention times matched those of the standard compounds within ± 0.1 min. Second, the signal-to-noise ratio was greater than 5:1. Third, the isotopic ratios for bromine ion pairs were within ± 15% of the theoretical values. In addition to the 20 blood samples, we also prepared 11 blank samples with pure water (~ 10 mL) as the blank matrix. Only 2,4-DBP (dibromophenol), 2,4,6-TBP (tribromo phenol), BDE-47, and BDE-99 were detected in the blank samples, and the blank values were around 9%, 7%, 2%, and 9% of the average concentration values measured in the blood samples, respectively. The recoveries (mean ± SE) of the surrogate standards were 99 ± 3 % for BDE-77, and 90 ± 6 % for 4-HO-^13^C_12_-PCB-187, respectively. In this article, the data were not blank or recovery corrected.

## Results

We measured PBDE congeners 28, 47, 99, 100, 153, and 154, which were the most abundant PBDE congeners observed in human blood in most other studies (Hites 2004). The mean and median concentrations of these six congeners ranged from 2.3 to 70 ng/g and from 0.8 to 13 ng/g lipid, respectively, in the fetal samples and from 0.5 to 17 ng/g and 0.3 to 15 ng/g lipid, respectively, in the maternal samples [Table 1; the full data set is given in the Supplemental Material (available online at http://www.ehponline.org/members/2008/11660/suppl.pdf)]. Among these congeners, BDE-28, 47, 99, and 100 were detected in all 20 samples, BDE-153 was detected in 19 samples, and BDE-154 was detected in 17 samples. No methoxylated PBDEs (MeO-PBDEs) were detected in the neutral clean-up fraction.

We also measured the concentrations of 18 potential phenolic metabolites, including 3 bromophenols and 15 hydroxylated PBDEs [see Supplemental Material (available online at http://www.ehponline.org/members/2008/11660/suppl.pdf) for the structures of these HO-PBDEs]. The 3 bromophenols were detected in all samples, and 7 of the 15 HO-PBDEs were identified and quantitated in all or some of the samples. The concentrations of these hydroxylated metabolites and their percent of the total phenolic compounds are shown in Table 1.

## Discussion

### PBDEs

Mazdai et al. (2003) reported that the concentrations of PBDEs in fetal blood did not differ from those in the corresponding maternal blood, and, based on analysis of unpaired maternal and fetal samples, our study confirms this finding [see Supplemental Material (available online at http://www.ehponline.org/members/2008/11660/suppl.pdf) for a statistical analysis comparing the fetal and maternal concentrations]. In the present study there was only one set of paired maternal–fetal samples; therefore, we could not directly determine if the HO-PBDE blood levels correlated between mother and baby. The fetal liver and adrenal glands express type I and type II enzymes capable of metabolizing xenobiotics that cross the placenta (Syme et al. 2004). Furthermore, the placenta expresses type I enzymes, and these are inducible by xenobiotics, such as those in cigarette smoke (Hakkola et al. 1998). Thus, the question of whether the fetus is at greater or lesser risk of exposure to PBDE metabolites requires further study.

In this study, the total concentrations of the 6 PBDE congeners in these 20 samples ranged from 4.7 to 800 ng/g lipid, with an average of 100 and a median of 31 ng/g lipid (Table 1). These values are close to previously reported PBDE concentrations in human blood from Indiana, where the concentrations ranged from 14 to 580 ng/g lipid (*n* = 24; Mazdai et al. 2003), and they are close to concentrations measured in fetal blood from Baltimore, Maryland (from not detected to 310 ng/g lipid for BDE-47, *n* = 297; Herbstman et al. 2007). All the concentrations reported here were much higher than those reported for human blood from Europe (e.g., 1.1–20 ng/g lipid for total PBDEs in 50 serum samples from Sweden and not detected to 6.1 ng/g lipid for BDE-47 in 81 maternal and fetal serum samples from the Netherlands) (Meijer et al. 2008; Weiss et al. 2006), indicating that North Americans are exposed to higher levels of PBDEs than are Europeans.

**Figure 1:**
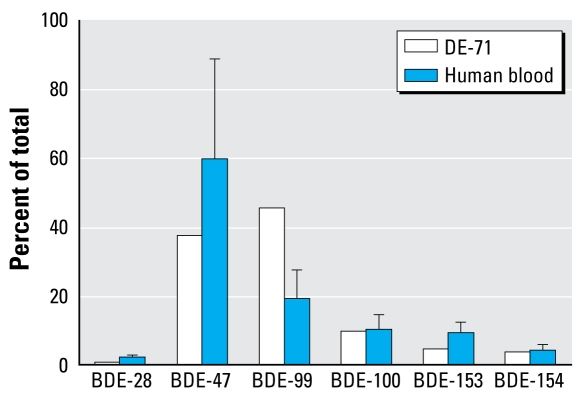
Congener profiles of PBDEs (percent of the total measured PBDEs) in human blood samples (mean ± SE, *n* = 20) and in a commercial penta-BDE product (DE-71).

The average profile of the PBDE congeners measured in this study is shown in Figure 1. For comparison, the congener profile of DE-71, an important commercial penta-BDE mixture and the presumptive source of PBDEs in human blood, is also shown.

Figure 1 shows that the percentage of BDE-99 in human blood was much lower than in DE-71, whereas the percentage of BDE-47 and 153 in human blood was higher than in DE-71. Neglecting the potential for slightly different uptake efficiencies of these congeners, these data suggest that, in human blood, BDE-99 may be the least persistent PBDE congener in DE-71. In fact, although BDE-99 is the most abundant congener of commercial DE-71, BDE-47 is usually the most abundant congener found in human blood samples (Hites 2004), and BDE-153 is sometimes the most abundant congener in people from low-exposure regions (Meijer et al. 2008; Weiss et al. 2006).

Different metabolic rates among the congeners might cause the different congener profiles observed for DE-71 and human blood. As found in this study, BDE-99 was more likely to be degraded to HO-PBDEs than were BDE-47 and BDE-100.

### Hydroxylated metabolites

**Figure 2:**
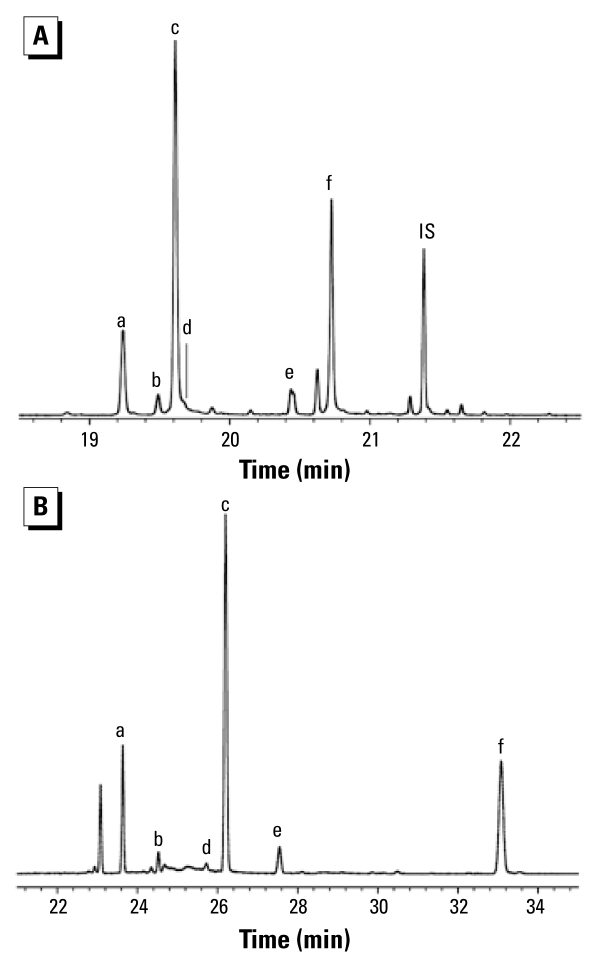
GC/MS (ECNI) chromatogram of methylated HO-PBDEs in a blood sample from the United States using (*A*) a nonpolar column (Rxi-5; 15 m) and (*B*) a polar column (SP-2331; 30 m). The identified target compounds were as follows: *a,* 6-HO-BDE-47; *b,* 3-HO-BDE-47; *c,* 5-HO-BDE-47; *d,* 4′-HO-BDE-49; *e,* 6′-HO-BDE-99; and *f,* 5′-HO-BDE-99. IS, internal standard (BDE-166). The bromophenols and 4-HO-^13^C_12_-PCB-187 are not shown.

In this study, hydroxylated metabolites included both mono-hydroxylated PBDEs (HO-PBDEs) and bromophenols. Of the 18 assessed hydroxylated metabolites, 7 HO-PBDEs and 3 bromophenols were measured in almost all of the 20 samples. Typical chromatograms of methylated HO-PBDEs on two different GC columns are shown in Figure 2.

One might assume that the hydroxylated metabolites, being more polar molecules, would be excreted more readily than the parent PBDEs, and thus, the concentration of these metabolites would be much lower than those of PBDEs in blood. This is not what we observed. The average total concentration of HO-PBDEs and bromophenols was 79 ng/g lipid (range, 2.0–900 ng/g lipid). The average concentration ratio of hydroxylated metabolites to PBDEs was 0.85 (range, 0.10–2.8), indicating that concentration of these metabolites was comparable to or even higher than that of PBDEs in these samples. This ratio was also much higher than that found in blood samples from young people from Managua, Nicaragua (Athanasiadou et al. 2008), perhaps because some abundant hydroxylated metabolites (5-HO-BDE-47, 6′-HO-BDE-99, and bromophenols) were not measured in that study. The high concentrations of metabolites and their relatively high ratio to PBDEs indicate that the hydroxylated metabolites of PBDEs may accumulate in human blood; and thus in this paper, we provide the concentrations of the hydroxylated metabolites on a lipid weight basis.

### HO-PBDEs

**Figure 3:**
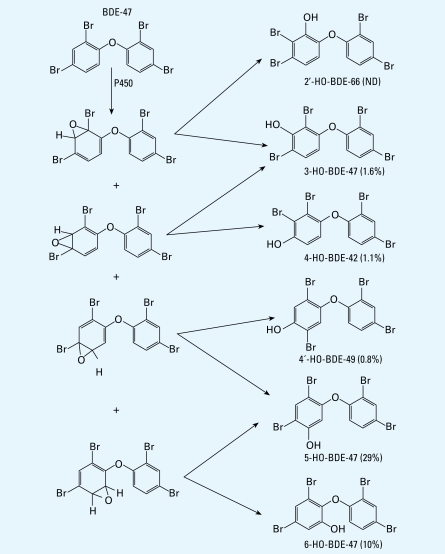
Proposed hydroxylation pathway of BDE-47 in humans and the percentage of metabolites (mean) in blood samples measured in this study. ND, not detected.

As discussed above, BDE-47 is an important congener in the commercial penta-BDE product and the most abundant PBDE congener found in human blood in this and in most other studies (Hites 2004). According to the proposed metabolic pathway for mice (Qiu et al. 2007), there are six possible monohydroxylated PBDE metabolites of BDE-47, presumably produced by cytochrome P450 enzymes. These metabolites are 3-HO-BDE-47, 5-HO-BDE-47, 6 - H O - B D E - 4 7, 4 - H O - B D E - 4 2, 4′-HO-BDE-49, and 2′-HO-BDE-66, the structures of which are shown in Figure 3. Note that the last three metabolites require a bromine shift via an arene oxide during the hydroxylation process.

In our previous study on mice, which were dosed with DE-71 at 45 mg/kg, 4-HO-BDE-42 was the most dominant metabolite of BDE-47 and accounted for 56% of the total HO-tetra-BDEs in mouse plasma, followed by 3-HO-BDE-47 (16%) and 4′-HO-BDE-49 (13%) (Qiu et al. 2007). The metabolite pro-file is very different in the human blood samples studied here. Although 4′-HO-BDE-49 and 4-HO-BDE-42 were detected in some of the 20 human blood samples, two metabolites formed without a bromine shift (5-HO-BDE-47 and 6-HO-BDE-47) were more abundant, especially 5-HO-BDE-47, which was not even detected in mice exposed to high doses of DE-71 (Qiu et al. 2007). As shown in Table 1 and in Figure 3, 5-HO-BDE-47 was the most abundant HO-tetra-BDE, followed by 6-HO-BDE-47. These two metabolites were detected in all 20 samples and accounted for 90% of the total HO-tetra-BDEs. 2′-HO-BDE-66 was not detected; 3-HO-BDE-47, 4′-HO-BDE-49 and 4-HO-BDE-42 were detected in some of the samples but at much lower concentrations.

The difference in the metabolic profile between humans and mice may be the result of species differences in cytochrome P450 enzyme expression. The superfamily of P450 has many subfamilies based on amino acid sequence identities (Nelson et al. 1993), and each different subfamily of P450 has a different selectivity in oxidation of the halogenated phenyl ring (Bogaards et al. 1995). Results of our study of PBDE metabolites in mice suggest that oxidative debromination occurred, and this accounts for the production of several *para*hydroxylated metabolites from BDE-47 (Qiu et al. 2007). The complex chemical reactions involved in oxidative dehalogenation (Isin and Guengerich 2007) are likely to require specific CYP enzymes. Although the precise set of CYP enzymes involved in oxidative dehalogenation in mammals is unknown, the lack of *para*hydroxylated metabolites in human serum is likely due to the different subfamily profiles in mice and humans (Bogaards et al. 2000). The *para*hydroxylated metabolites are likely to behave as endocrine disruptors (Hamers et al. 2008; Meerts et al. 2000; Mercado-Feliciano and Bigsby 2008a), and therefore the lack of these in humans may explain the lack of any correlation between PBDE and thyroid hormone concentrations in blood of mothers and their babies (Mazdai et al. 2003). However, the enzyme profiles of the placenta and fetal liver change throughout the course of pregnancy (Hakkola et al. 1998), and there may be transient differences in the PBDE metabolite profiles as a result. Furthermore, placental CYP1A1 is highly inducible by cigarette smoke (Hakkola et al. 1998). The potential for transient differences in PBDE metabolite profiles during the course of pregnancy and for a correlation between exposure to tobacco smoke and metabolism require further study.

Some HO-PBDEs are more toxic because of the specific position of hydroxyl group; for example, 4-HO-BDE-42, 4′-HO-BDE-49, and 3-HO-BDE-47 were shown to have about four times stronger affinity to transthyretin than thyroxin (Hamers et al. 2008). Our data indicate that human P450 enzymes do not produce many of these toxic isomers. However, 5-HO-BDE-47 has a three times stronger affinity to transthyretin than thyroxin (Hamers et al. 2008). Given the concentration of 8.7 nM free thyroxin in serum during pregnancy reported by Sterling and Hegedus (1962) (compared with the average concentration of 5-HO-BDE-47 of 0.1 nmol/kg plasma, with the highest concentration being 1.2 nmol/kg plasma in the present study), these metabolites, especially 5-HO-BDE-47, might have substantial human effects because of their relatively high concentrations in blood.

BDE-99 is the most abundant congener of DE-71; however, as discussed above, concentrations of BDE-99 were lower than that of BDE-47 in human blood in this and in most other studies, perhaps because BDE-99 was converted to hydroxylated PBDEs. Like the metabolite pattern of BDE-47, 5′-HO-BDE-99 was the most abundant hydroxylated metabolite of BDE-99, followed by 6′-HO-BDE-99 (Table 1 and Figure 2). The ratio of 5′-HO-BDE-99 + 6′-HO-BDE-99 to 5-HO-BDE-47 + 6-HO-BDE-47 was 0.84 on average (range, 0.24–3.2), which was significantly higher than the ratio of BDE-99 to BDE-47 (0.39 on average; range, 0.17–0.69; *p* < 0.01, *t*-test). Given the symmetrical structure of BDE-47, it has a higher probability to form 5-HO-BDE-47 and 6-HO-BDE-47 than BDE-99 has to form 5′-HO-BDE-99 and 6′-HO-BDE-99. Thus, we conclude that BDE-99 was more likely than BDE-47 to be hydroxylated. This may also explain why the concentration of BDE-99 was usually lower than that of BDE-47 in human blood, although it was more abundant in the commercial penta-BDE mixtures.

As discussed above, in human blood BDE-47 and BDE-99 have similar metabolic profiles, and hydroxylation mainly occurs on the phenyl ring with two bromines. This process resulted in 5-HO-BDE-47 and 5′-HO-BDE-99 as the two most abundant metabolites of BDE-47 and BDE-99, respectively. If this process were true for BDE-100, we might expect that 5′-HO-BDE-100 should be the most important hydroxylated metabolite of BDE-100; however, 5′-HO-BDE-100 was not detected in this study. In fact, there were no other large GC peaks on either column in the retention time regions where hydroxylated penta-BDEs might be expected to elute (Figure 2). The lack of detection of 5′-HO-BDE-100 suggests that BDE-100 is more resistant to hydroxylation than is BDE-99, explaining the increasing ratio of BDE-100 to BDE-99 from commercial DE-71 (0.21) to human blood (0.59) (Figure 1).

In the present study, we did not detect 5′-HO-BDE-100 or several other HO-penta-BDEs, including 4-HO-BDE-90, 4′-HO-BDE-101, and 4′-HO-BDE-103. Theoretically, these *para*-HO-penta-BDEs could be formed from BDE-99 or BDE-100 via hydroxylation with a bromine shift; apparently however, this bromine shift was not caused by human P450 enzymes. We should note that 4-HO-BDE-90 has the same retention time as 5′-HO-BDE-99 on nonpolar GC columns (such as Rxi-5 and DB-5); however, on polar columns (such as SP-2331) these two compounds can easily be distinguished. In another study, Athanasiadou et al. (2008) quantified this peak relative to 4-HO-BDE-90 with a DB-5 column.

### Bromophenols

In our previous study of mice (Qiu et al. 2007), three bromophenols were identified in blood samples after exposure to DE-71. These bromophenols were also detected in human blood samples. To our knowledge, 2,4,5-TBP is not a commercial product, thus the presence of 2,4,5-TBP in human blood (at 6.4 ng/g lipid) indicates that the diphenyl ether bond of some PBDE congeners (BDE-99 for example) can be cleaved. For 2,4-DBP and 2,4,6-TBP, although they were also detected in blanks in this and in other studies (Thomsen et al. 2001), the concentrations of these two compounds in the blood samples (averaging 16 and 4.6 ng/g lipid for 2,4-DBP and 2,4,6-TBP, respectively) were > 10-fold higher than those measured in the blanks, indicating that these two bromophenols came from sources other than the laboratory blank. One source of these two contaminants might be exposure to these chemicals because both 2,4-DBP and 2,4,6-TBP have been used as flame retardants (World Health Organization 1997). Another source, such as 2,4,5-TBP, might be cleavage of the diphenyl ether bond of PBDEs. Based on the proposed metabolic pathway for mice (Qiu et al. 2007), 2,4-DBP could be a metabolite of BDE-47, and 2,4,6-TBP could be a metabolite of BDE-100 and BDE-154.

The average total concentration of the three bromophenols was 27 ng/g lipid (range, 1.2–310 ng/g lipid). For comparison, the average total concentration of the measured HO-PBDEs was 52 ng/g lipid (range, 0.8–590 ng/g lipid). Thus, although the bromophenols might have other sources, the concentrations of bromophenols were comparable to those of HO-PBDEs, suggesting that cleavage of the diphenyl ether bond was an important metabolic pathway for PBDEs in humans.

## Summary

Unlike the metabolites measured in mice after dosing with PBDE, the HO-PBDE metabolites of BDE-47 in humans without a bromine shift were abundant. Two metabolites, 5-HO-BDE-47 and 6-HO-BDE-47, accounted for 90% of the total HO-tetra-BDE concentration in our subjects. 5′-HO-BDE-99 and 6′-HO-BDE-99 were the most abundant metabolites of BDE-99 in our subjects. The relative concentrations between precursor and the corresponding HO-PBDE indicated that BDE-99 was more likely to be hydroxylated than BDE-47 and BDE-100, and this observation may explain the different congener profiles noticed for PBDEs in human blood as opposed to the commercial penta-BDE mixture. In addition to HO-PBDEs, three bromophenols were also detected in human blood, indicating the cleavage of the diphenyl ether bond of PBDEs. The total concentrations of the hydroxylated metabolites (HO-PBDEs and bromophenols) were close to those of the PBDEs, suggesting that these hydroxylated metabolites may be accumulating in human blood.

## Figures and Tables

**Table 1:** Concentrations (ng/g lipid) of PBDE and HO-PBDE congeners in fetal and maternal blood and their percent of total measurable analytes.

	Fetal samples (*n* = 16)			Maternal samples (*n* = 4)			Combined samples ( *n* = 20)			
Compound or congener	Mean ± SE	Median	% of total mean	Mean ± SE	Median	% of total mean	Mean ± SE	Median	% of total mean	Mice (*n* = 15)^a^ % of total mean
PBDE congener										
BDE-28	2.3 ± 1.0	0.8	1.9	0.5 ± 0.2	0.3	1.4	1.9 ± 0.8	0.7	1.9	0.2
BDE-47	70 ± 36	13	59	17 ± 5.1	15	49	60 ± 29	13	58	19
BDE-99	22 ± 11	5.3	18	6.3 ± 1.9	6.3	18	19 ± 8.6	5.3	18	20
BDE-100	12 ± 5.5	2.6	9.8	3.0 ± 0.3	3.0	8.7	9.9 ± 4.4	2.6	9.7	6.6
BDE-153	9.4 ± 4.1	2.7	8.0	6.7 ± 2.5	5.5	20	8.9 ± 3.2	3.8	8.7	54
BDE-154	4.6 ± 2.4	1.3	3.9	1.2 ± 0.1	1.2	3.6	3.8 ± 1.9	1.2	3.7	1.0
Total	120 ± 55	31		34 ± 8.5	34		100 ± 45	31		
Phenolic metabolite										
2,4-DBP	20 ± 12	4.7	21	1.5 ± 0.5	1.3	21	16 ± 9.6	3.5	21	15
2,4,5-TBP	7.9 ± 6.0	1.2	8.2	0.2 ± 0.03	0.2	3.6	6.4 ± 4.8	0.7	8.1	16
2,4,6-TBP	5.6 ± 1.3	5.1	5.7	0.8 ± 0.3	0.6	12	4.6 ± 1.1	3.0	5.9	1.1
4′-HO-BDE-17	ND	ND	ND	ND	ND	ND	ND	ND	ND	3.5
2′-HO-BDE-28	ND	ND	ND	ND	ND	ND	ND	ND	ND	2.3
4-HO-BDE-42	0.9 ± 0.3	ND	0.9	ND	ND	ND	0.9 ± 0.3	ND	1.1	38
3-HO-BDE-47	1.6 ± 1.1	0.4	1.7	0.1 ± 0.02	0.09	1.3	1.3 ± 0.8	0.3	1.6	11
5-HO-BDE-47	28 ± 16	5.7	29	1.6 ± 0.5	1.4	23	23 ± 13	4.2	29	ND
6-HO-BDE-47	9.9 ± 4.9	1.0	10	0.3 ± 0.1	0.2	3.9	7.9 ± 4.0	0.8	10	4.6
4′-HO-BDE-49	0.9 ± 0.2	ND	0.9	0.3 ± 0.06	0.3	3.5	0.7 ± 0.2	ND	0.8	8.7
5′-HO-BDE-99	22 ± 15	4.3	23	2.0 ± 0.6	1.7	28	18 ± 12	3.1	23	ND
6′-HO-BDE-99	1.9 ± 1.0	0.6	1.9	0.3 ± 0.04	0.3	3.7	1.5 ± 0.8	0.4	1.9	ND
Total	97 ± 55	22		7.0 ± 1.7	6.3		79 ± 44	19		

ND, not detected.

a Data from Qiu et al. (2007).
